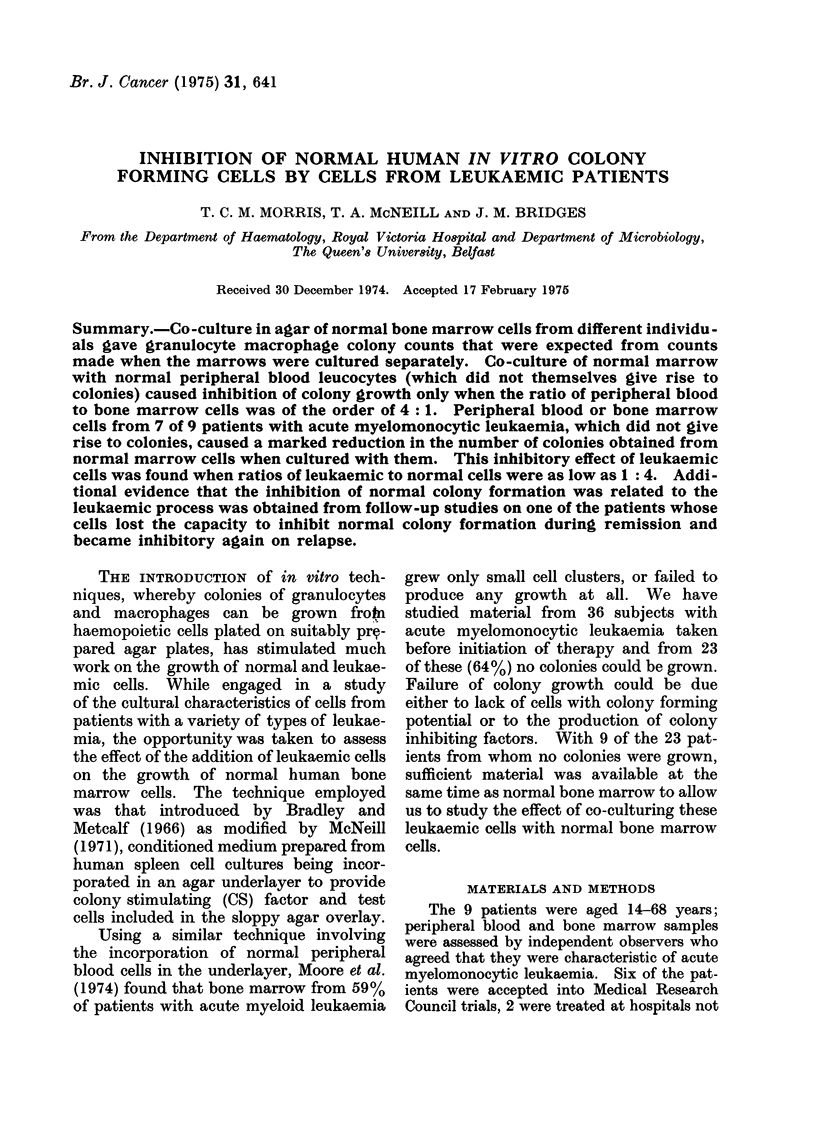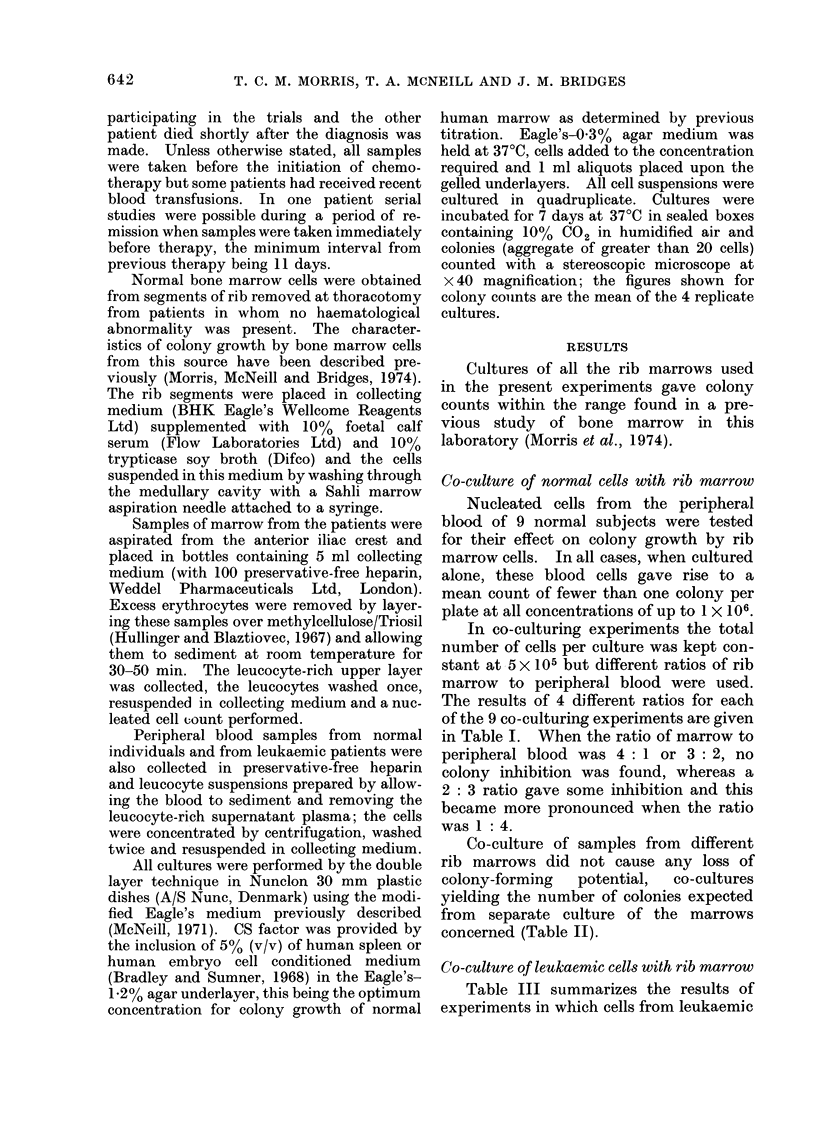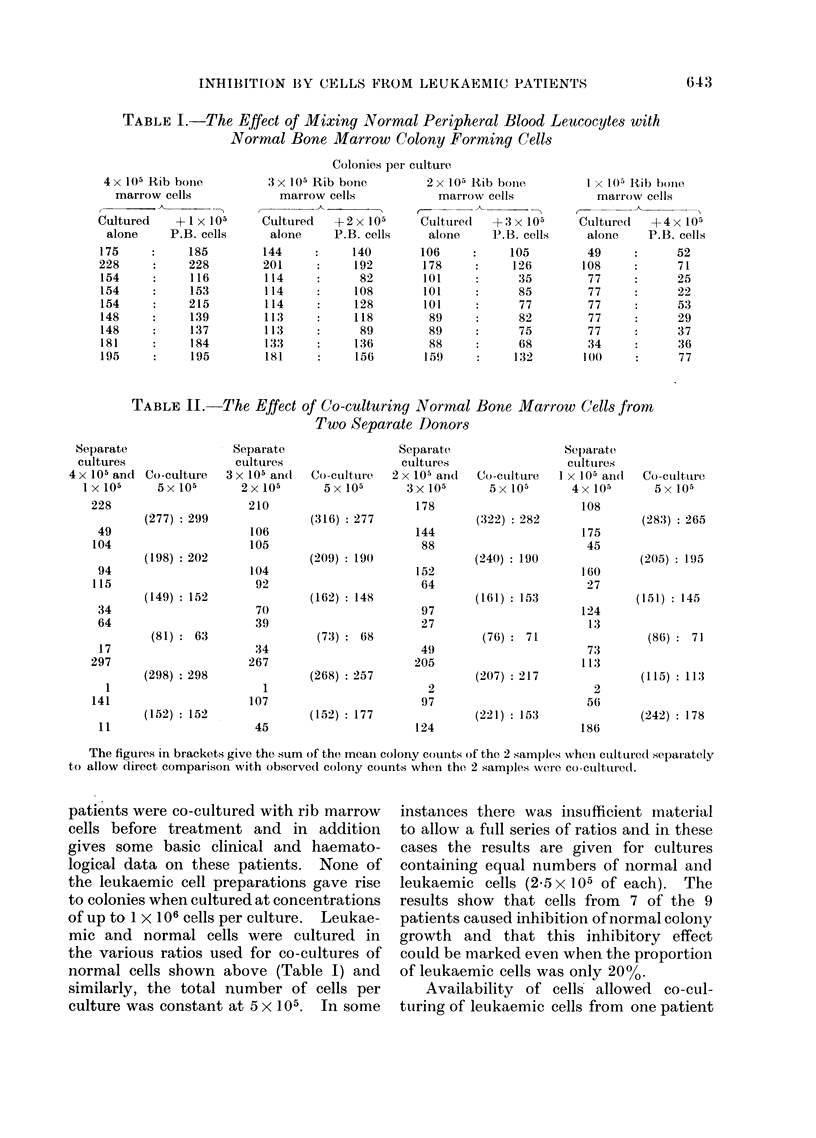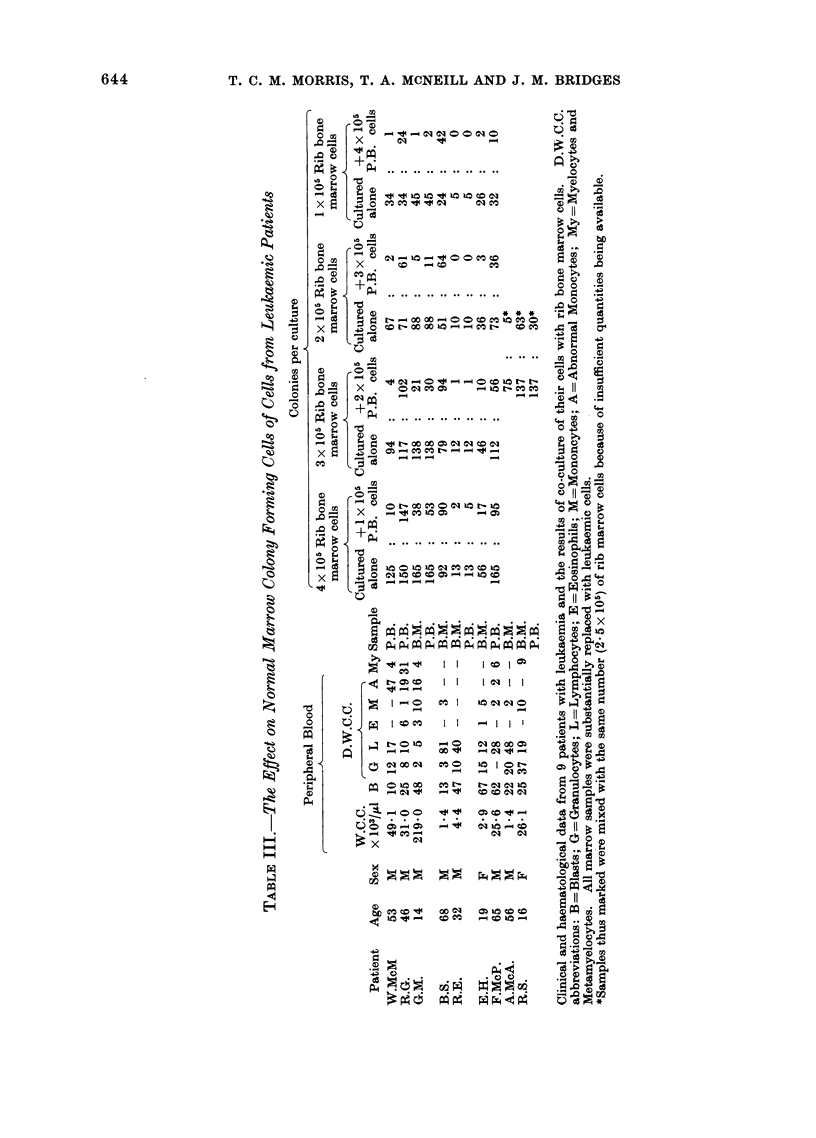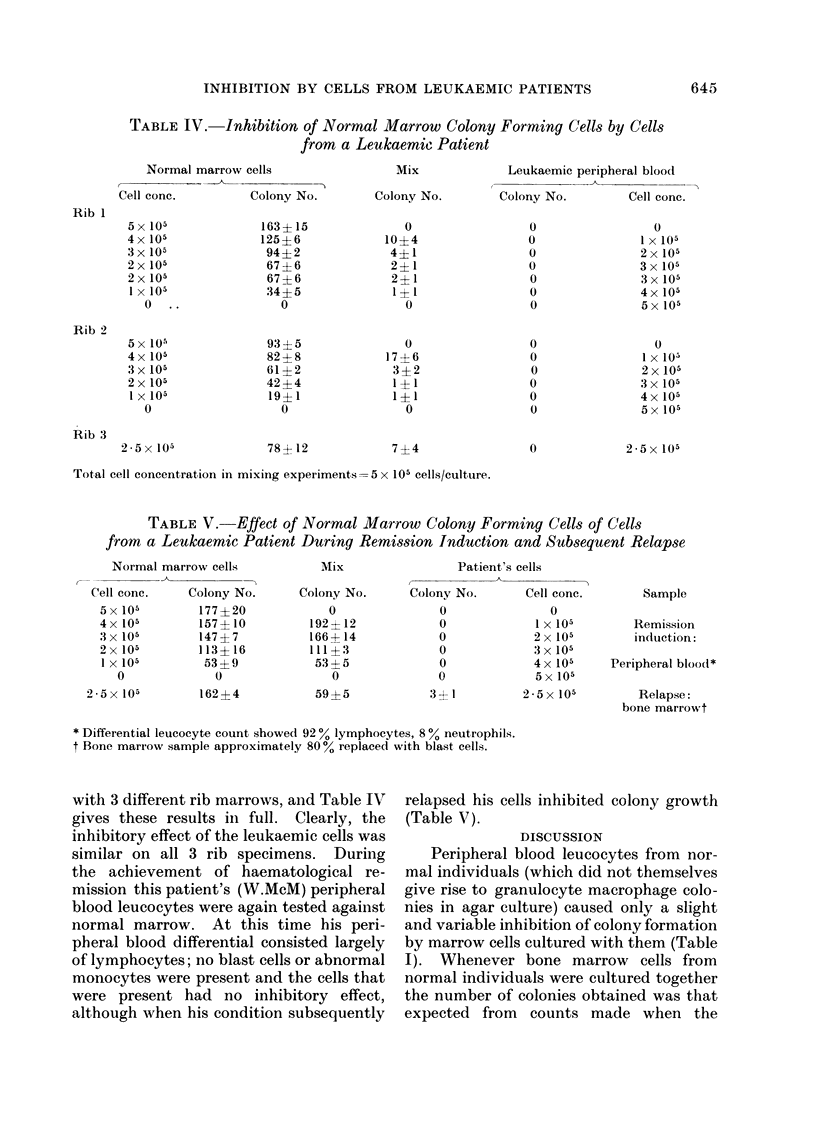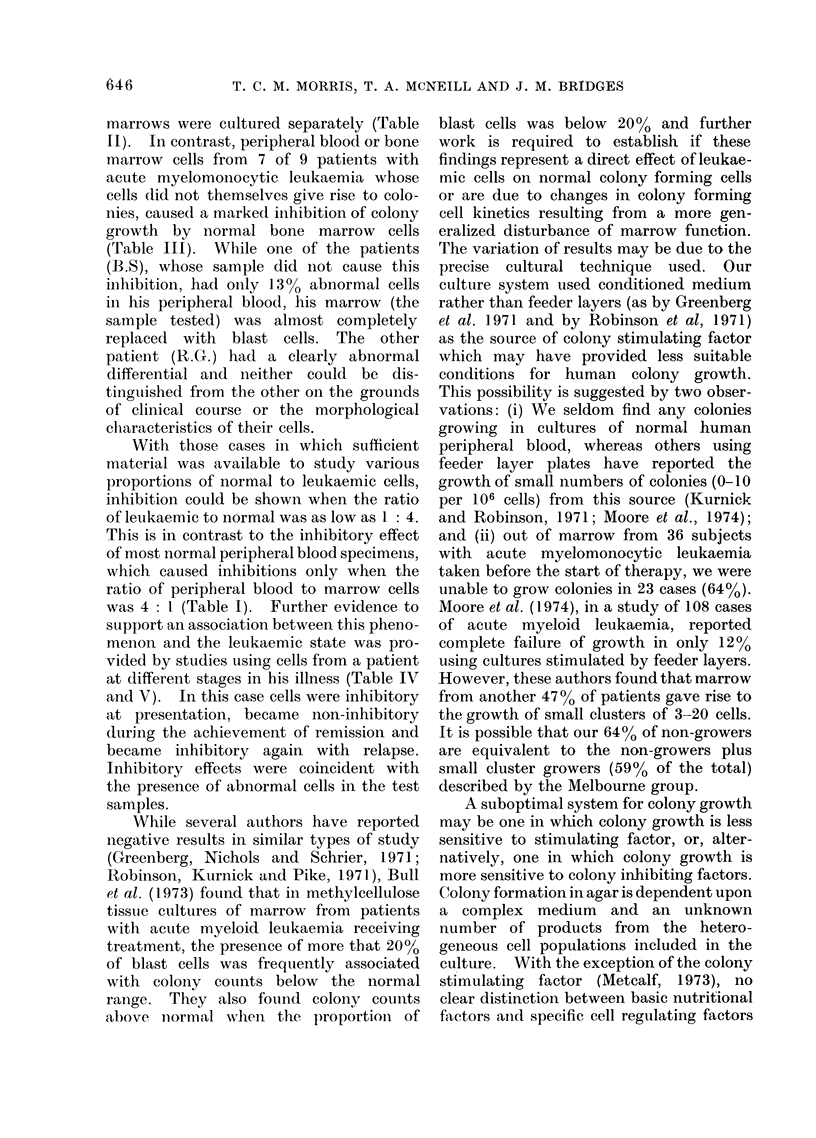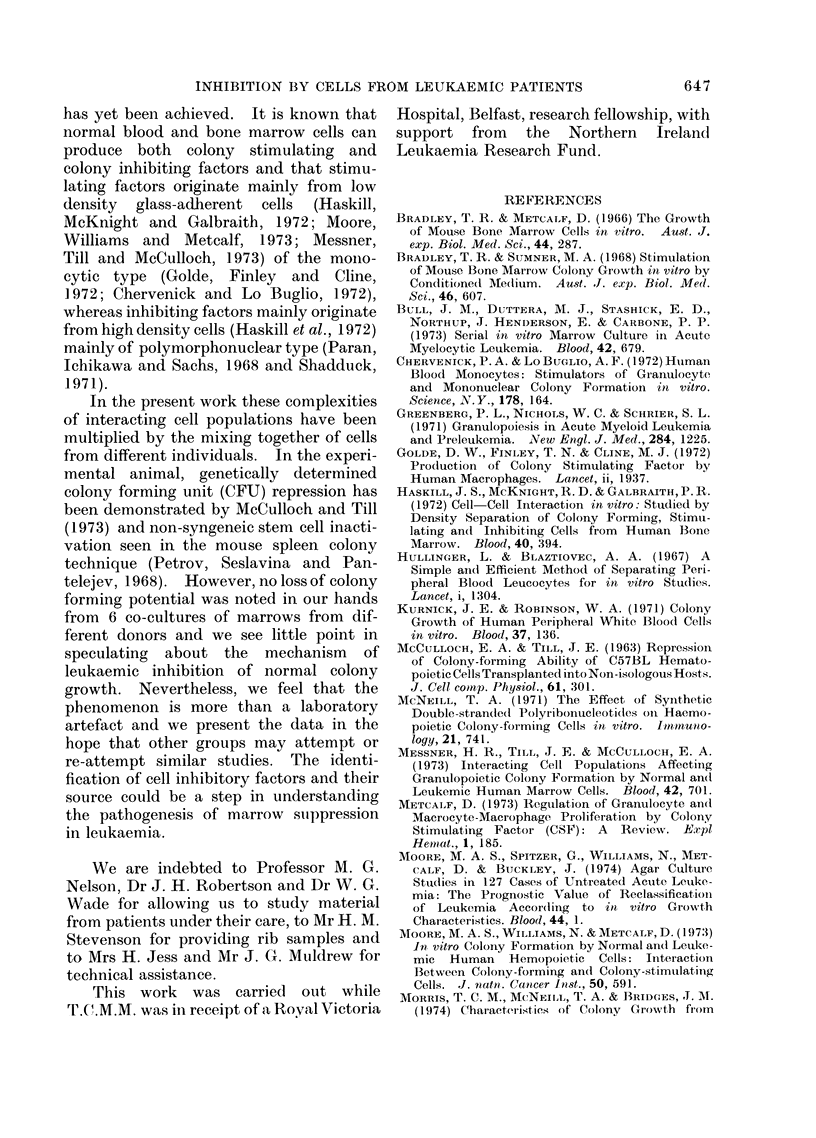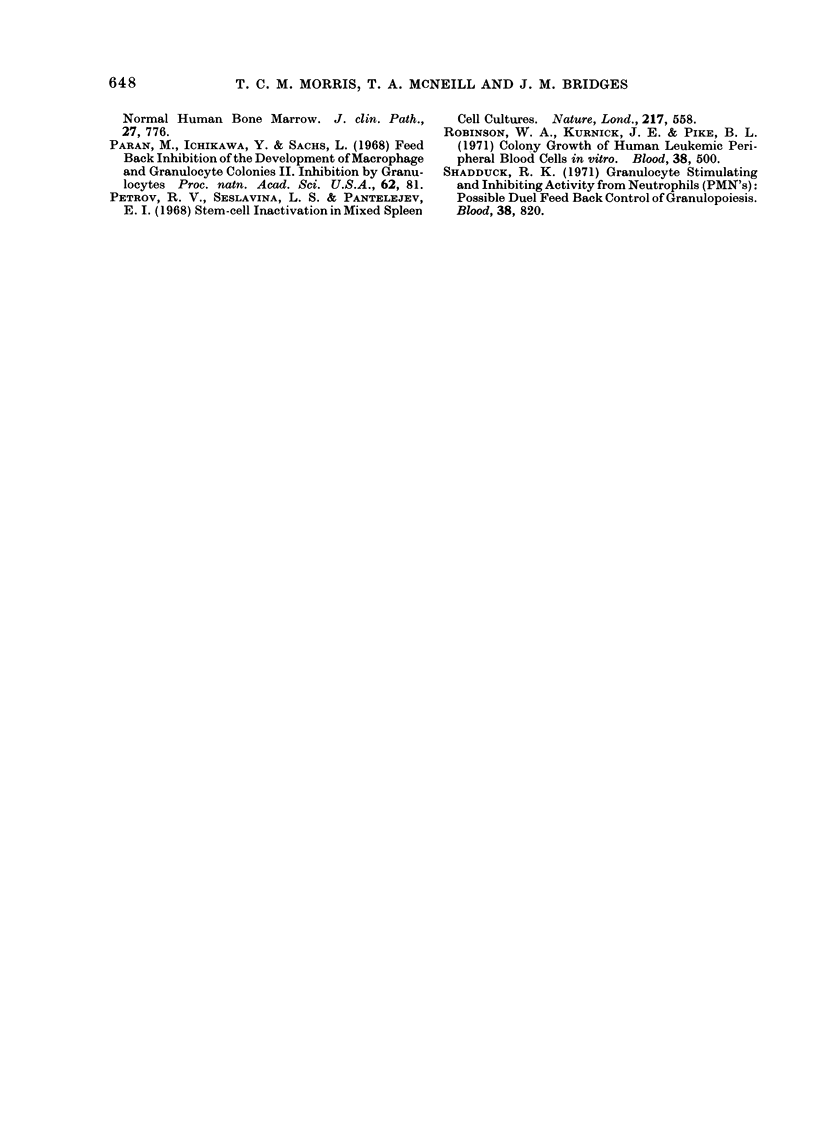# Inhibition of normal human in vitro colony forming cells by cells from leukaemic patients.

**DOI:** 10.1038/bjc.1975.110

**Published:** 1975-06

**Authors:** T. C. Morris, T. A. McNeill, J. M. Bridges

## Abstract

Co-culture in agar of normal bone marrow cells from different individuals gave granulocyte macrophage colony counts that were expected from counts made when the marrows were cultured separately. Co-culture of normal marrow with normal peripheral blood leucocytes (which did not themselves give rise to colonies) caused inhibition of colony growth only when the ratio of peripheral blood to bone marrow cells was of the order of 4 : 1. Peripheral blood or bone marrow cells from 7 of 9 patients with acute myelomonocytic leukaemia, which did not give rise to colonies, caused a marked reduction in the number of colonies obtained from normal marrow cells when cultured with them. This inhibitory effect of leukaemic cells was found when ratios of leukaemic to normal cells were as low as 1 : 4. Additional evidence that the inhibition of normal colony formation was related to the leukaemic process was obtained from follow-up studies on one of the patients whose cells lost the capacity to inhibit normal colony formation during remission and became inhibitory again on relapse.


*Br. J. Cancer* (1975) **31**, 641

### INHIBITION OF NORMAL HUMAN *IN VITRO* COLONY FORMING CELLS BY CELLS FROM LEUKAEMIC PATIENTS

T. C. M. MORRIS, T. A. McNEILL AND J. M. BRIDGES

*From the Department of Haematology, Royal Victoria Hospital and Department of Microbiology, The Queen's University, Belfast*

Received 30 December 1974. Accepted 17 February 1975

**Summary.—Co-culture in agar of normal bone marrow cells from different individuals gave granulocyte macrophage colony counts that were expected from counts made when the marrows were cultured separately. Co-culture of normal marrow with normal peripheral blood leucocytes (which did not themselves give rise to colonies) caused inhibition of colony growth only when the ratio of peripheral blood to bone marrow cells was of the order of 4 : 1. Peripheral blood or bone marrow cells from 7 of 9 patients with acute myelomonocytic leukaemia, which did not give rise to colonies, caused a marked reduction in the number of colonies obtained from normal marrow cells when cultured with them. This inhibitory effect of leukaemic cells was found when ratios of leukaemic to normal cells were as low as 1 : 4. Additional evidence that the inhibition of normal colony formation was related to the leukaemic process was obtained from follow-up studies on one of the patients whose cells lost the capacity to inhibit normal colony formation during remission and became inhibitory again on relapse.**

THE INTRODUCTION of *in vitro* techniques, whereby colonies of granulocytes and macrophages can be grown from haemopoietic cells plated on suitably prepared agar plates, has stimulated much work on the growth of normal and leukaemic cells. While engaged in a study of the cultural characteristics of cells from patients with a variety of types of leukaemia, the opportunity was taken to assess the effect of the addition of leukaemic cells on the growth of normal human bone marrow cells. The technique employed was that introduced by Bradley and Metcalf (1966) as modified by McNeill (1971), conditioned medium prepared from human spleen cell cultures being incorporated in an agar underlayer to provide colony stimulating (CS) factor and test cells included in the sloppy agar overlay.

Using a similar technique involving the incorporation of normal peripheral blood cells in the underlayer, Moore *et al.* (1974) found that bone marrow from 59% of patients with acute myeloid leukaemia grew only small cell clusters, or failed to produce any growth at all. We have studied material from 36 subjects with acute myelomonocytic leukaemia taken before initiation of therapy and from 23 of these (64%) no colonies could be grown. Failure of colony growth could be due either to lack of cells with colony forming potential or to the production of colony inhibiting factors. With 9 of the 23 patients from whom no colonies were grown, sufficient material was available at the same time as normal bone marrow to allow us to study the effect of co-culturing these leukaemic cells with normal bone marrow cells.

### MATERIALS AND METHODS

The 9 patients were aged 14–68 years; peripheral blood and bone marrow samples were assessed by independent observers who agreed that they were characteristic of acute myelomonocytic leukaemia. Six of the patients were accepted into Medical Research Council trials, 2 were treated at hospitals not participating in the trials and the other patient died shortly after the diagnosis was made. Unless otherwise stated, all samples were taken before the initiation of chemotherapy but some patients had received recent blood transfusions. In one patient serial studies were possible during a period of remission when samples were taken immediately before therapy, the minimum interval from previous therapy being 11 days.

Normal bone marrow cells were obtained from segments of rib removed at thoracotomy from patients in whom no haematological abnormality was present. The characteristics of colony growth by bone marrow cells from this source have been described previously (Morris, McNeill and Bridges, 1974). The rib segments were placed in collecting medium (BHK Eagle's Wellcome Reagents Ltd) supplemented with 10% foetal calf serum (Flow Laboratories Ltd) and 10% trypticase soy broth (Difco) and the cells suspended in this medium by washing through the medullary cavity with a Sahli marrow aspiration needle attached to a syringe.

Samples of marrow from the patients were aspirated from the anterior iliac crest and placed in bottles containing 5 ml collecting medium (with 100 preservative-free heparin, Weddel Pharmaceuticals Ltd, London). Excess erythrocytes were removed by layering these samples over methylcellulose/Triosil (Hullinger and Blaztiovec, 1967) and allowing them to sediment at room temperature for 30–50 min. The leucocyte-rich upper layer was collected, the leucocytes washed once, resuspended in collecting medium and a nucleated cell count performed.

Peripheral blood samples from normal individuals and from leukaemic patients were also collected in preservative-free heparin and leucocyte suspensions prepared by allowing the blood to sediment and removing the leucocyte-rich supernatant plasma; the cells were concentrated by centrifugation, washed twice and resuspended in collecting medium.

All cultures were performed by the double layer technique in Nunclon 30 mm plastic dishes (A/S Nunc, Denmark) using the modified Eagle's medium previously described (McNeill, 1971). CS factor was provided by the inclusion of 5% (v/v) of human spleen or human embryo cell conditioned medium (Bradley and Sumner, 1968) in the Eagle's–1·2% agar underlayer, this being the optimum concentration for colony growth of normal human marrow as determined by previous titration. Eagle's–0·3% agar medium was held at 37°C, cells added to the concentration required and 1 ml aliquots placed upon the gelled underlayers. All cell suspensions were cultured in quadruplicate. Cultures were incubated for 7 days at 37°C in sealed boxes containing 10% $CO_2$ in humidified air and colonies (aggregate of greater than 20 cells) counted with a stereoscopic microscope at ×40 magnification; the figures shown for colony counts are the mean of the 4 replicate cultures.

### RESULTS

Cultures of all the rib marrows used in the present experiments gave colony counts within the range found in a previous study of bone marrow in this laboratory (Morris *et al.*, 1974).

### *Co-culture of normal cells with rib marrow*

Nucleated cells from the peripheral blood of 9 normal subjects were tested for their effect on colony growth by rib marrow cells. In all cases, when cultured alone, these blood cells gave rise to a mean count of fewer than one colony per plate at all concentrations of up to $1 \times 10^6$.

In co-culturing experiments the total number of cells per culture was kept constant at $5 \times 10^5$ but different ratios of rib marrow to peripheral blood were used. The results of 4 different ratios for each of the 9 co-culturing experiments are given in Table I. When the ratio of marrow to peripheral blood was 4 : 1 or 3 : 2, no colony inhibition was found, whereas a 2 : 3 ratio gave some inhibition and this became more pronounced when the ratio was 1 : 4.

Co-culture of samples from different rib marrows did not cause any loss of colony-forming potential, co-cultures yielding the number of colonies expected from separate culture of the marrows concerned (Table II).

### *Co-culture of leukaemic cells with rib marrow*

Table III summarizes the results of experiments in which cells from leukaemic

TABLE I.—*The Effect of Mixing Normal Peripheral Blood Leucocytes with Normal Bone Marrow Colony Forming Cells*

Colonies per culture

| $4 \times 10^5$ Rib bone marrow cells | | $3 \times 10^5$ Rib bone marrow cells | | $2 \times 10^5$ Rib bone marrow cells | | $1 \times 10^5$ Rib bone marrow cells | |
|---|---|---|---|---|---|---|---|
| Cultured alone | $+1 \times 10^5$ P.B. cells | Cultured alone | $+2 \times 10^5$ P.B. cells | Cultured alone | $+3 \times 10^5$ P.B. cells | Cultured alone | $+4 \times 10^5$ P.B. cells |
| 175 : | 185 | 144 : | 140 | 106 : | 105 | 49 : | 52 |
| 228 : | 228 | 201 : | 192 | 178 : | 126 | 108 : | 71 |
| 154 : | 116 | 114 : | 82 | 101 : | 35 | 77 : | 25 |
| 154 : | 153 | 114 : | 108 | 101 : | 85 | 77 : | 22 |
| 154 : | 215 | 114 : | 128 | 101 : | 77 | 77 : | 53 |
| 148 : | 139 | 113 : | 118 | 89 : | 82 | 77 : | 29 |
| 148 : | 137 | 113 : | 89 | 89 : | 75 | 77 : | 37 |
| 181 : | 184 | 133 : | 136 | 88 : | 68 | 34 : | 36 |
| 195 : | 195 | 181 : | 156 | 159 : | 132 | 100 : | 77 |

TABLE II.—*The Effect of Co-culturing Normal Bone Marrow Cells from Two Separate Donors*

| Separate cultures $4 \times 10^5$ and $1 \times 10^5$ | Co-culture $5 \times 10^5$ | Separate cultures $3 \times 10^5$ and $2 \times 10^5$ | Co-culture $5 \times 10^5$ | Separate cultures $2 \times 10^5$ and $3 \times 10^5$ | Co-culture $5 \times 10^5$ | Separate cultures $1 \times 10^5$ and $4 \times 10^5$ | Co-culture $5 \times 10^5$ |
|---|---|---|---|---|---|---|---|
| 228 | | 210 | | 178 | | 108 | |
| | (277) : 299 | | (316) : 277 | | (322) : 282 | | (283) : 265 |
| 49 | | 106 | | 144 | | 175 | |
| 104 | | 105 | | 88 | | 45 | |
| | (198) : 202 | | (209) : 190 | | (240) : 190 | | (205) : 195 |
| 94 | | 104 | | 152 | | 160 | |
| 115 | | 92 | | 64 | | 27 | |
| | (149) : 152 | | (162) : 148 | | (161) : 153 | | (151) : 145 |
| 34 | | 70 | | 97 | | 124 | |
| 64 | | 39 | | 27 | | 13 | |
| | (81) : 63 | | (73) : 68 | | (76) : 71 | | (86) : 71 |
| 17 | | 34 | | 49 | | 73 | |
| 297 | | 267 | | 205 | | 113 | |
| | (298) : 298 | | (268) : 257 | | (207) : 217 | | (115) : 113 |
| 1 | | 1 | | 2 | | 2 | |
| 141 | | 107 | | 97 | | 56 | |
| | (152) : 152 | | (152) : 177 | | (221) : 153 | | (242) : 178 |
| 11 | | 45 | | 124 | | 186 | |

The figures in brackets give the sum of the mean colony counts of the 2 samples when cultured separately to allow direct comparison with observed colony counts when the 2 samples were co-cultured.

patients were co-cultured with rib marrow cells before treatment and in addition gives some basic clinical and haematological data on these patients. None of the leukaemic cell preparations gave rise to colonies when cultured at concentrations of up to $1 \times 10^6$ cells per culture. Leukaemic and normal cells were cultured in the various ratios used for co-cultures of normal cells shown above (Table I) and similarly, the total number of cells per culture was constant at $5 \times 10^5$. In some instances there was insufficient material to allow a full series of ratios and in these cases the results are given for cultures containing equal numbers of normal and leukaemic cells ($2 \cdot 5 \times 10^5$ of each). The results show that cells from 7 of the 9 patients caused inhibition of normal colony growth and that this inhibitory effect could be marked even when the proportion of leukaemic cells was only 20%.

Availability of cells allowed co-culturing of leukaemic cells from one patient

TABLE III.—*The Effect on Normal Marrow Colony Forming Cells of Cells from Leukaemic Patients*

| | | | Peripheral Blood | | | | | | | | | Colonies per culture | | | | | | | |
|---|---|---|---|---|---|---|---|---|---|---|---|---|---|---|---|---|---|---|---|
| | | | | D.W.C.C. | | | | | | | | $4\times10^5$ Rib bone marrow cells | | $3\times10^5$ Rib bone marrow cells | | $2\times10^5$ Rib bone marrow cells | | $1\times10^5$ Rib bone marrow cells | |
| Patient | Age | Sex | W.C.C. $\times10^3/\mu l$ | B | G | L | E | M | A | My | Sample | Cultured alone | $+1\times10^5$ P.B. cells | Cultured alone | $+2\times10^5$ P.B. cells | Cultured alone | $+3\times10^5$ P.B. cells | Cultured alone | $+4\times10^5$ P.B. cells |
| W.McM | 53 | M | 49·1 | 10 | 12 | 17 | – | – | 47 | 4 | P.B. | 125 | 10 | 94 | 4 | 67 | 2 | 34 | 1 |
| R.G. | 46 | M | 31·0 | 25 | 8 | 10 | 6 | 1 | 19 | 31 | P.B. | 150 | 147 | 117 | 102 | 71 | 61 | 34 | 24 |
| G.M. | 14 | M | 219·0 | 48 | 2 | 5 | 3 | 10 | 16 | 4 | B.M. | 165 | 38 | 138 | 21 | 88 | 5 | 45 | 1 |
| | | | | | | | | | | | P.B. | 165 | 53 | 138 | 30 | 88 | 11 | 45 | 2 |
| B.S. | 68 | M | 1·4 | 13 | 3 | 81 | – | 3 | – | – | B.M. | 92 | 90 | 79 | 94 | 51 | 64 | 24 | 42 |
| R.E. | 32 | M | 4·4 | 47 | 10 | 40 | – | – | – | – | B.M. | 13 | 2 | 12 | 1 | 10 | 0 | 5 | 0 |
| | | | | | | | | | | | P.B. | 13 | 5 | 12 | 1 | 10 | 0 | 5 | 0 |
| E.H. | 19 | F | 2·9 | 67 | 15 | 12 | 1 | 5 | – | – | B.M. | 56 | 17 | 46 | 10 | 36 | 3 | 26 | 2 |
| F.McP. | 65 | M | 25·6 | 62 | – | 28 | – | 2 | 2 | 6 | P.B. | 165 | 95 | 112 | 56 | 73 | 36 | 32 | 10 |
| A.McA. | 56 | M | 1·4 | 22 | 20 | 48 | – | 2 | – | – | B.M. | | | | 75 | 5* | | | |
| R.S. | 16 | F | 26·1 | 25 | 37 | 19 | - | 10 | – | 9 | B.M. | | | | 137 | 63* | | | |
| | | | | | | | | | | | P.B. | | | | 137 | 30* | | | |

Clinical and haematological data from 9 patients with leukaemia and the results of co-culture of their cells with rib bone marrow cells. D.W.C.C. abbreviations: B=Blasts; G=Granulocytes; L=Lymphocytes; E=Eosinophils; M=Mononcytes; A=Abnormal Monocytes; My=Myelocytes and Metamyelocytes. All marrow samples were substantially replaced with leukaemic cells.

*Samples thus marked were mixed with the same number ($2\cdot5\times10^5$) of rib marrow cells because of insufficient quantities being available.

TABLE IV.—*Inhibition of Normal Marrow Colony Forming Cells by Cells from a Leukaemic Patient*

| | Normal marrow cells | | Mix | Leukaemic peripheral blood | |
|---|---|---|---|---|---|
| | Cell conc. | Colony No. | Colony No. | Colony No. | Cell conc. |
| Rib 1 | | | | | |
| | $5\times10^5$ | $163\pm15$ | 0 | 0 | 0 |
| | $4\times10^5$ | $125\pm6$ | $10\pm4$ | 0 | $1\times10^5$ |
| | $3\times10^5$ | $94\pm2$ | $4\pm1$ | 0 | $2\times10^5$ |
| | $2\times10^5$ | $67\pm6$ | $2\pm1$ | 0 | $3\times10^5$ |
| | $2\times10^5$ | $67\pm6$ | $2\pm1$ | 0 | $3\times10^5$ |
| | $1\times10^5$ | $34\pm5$ | $1\pm1$ | 0 | $4\times10^5$ |
| | 0 .. | 0 | 0 | 0 | $5\times10^5$ |
| Rib 2 | | | | | |
| | $5\times10^5$ | $93\pm5$ | 0 | 0 | 0 |
| | $4\times10^5$ | $82\pm8$ | $17\pm6$ | 0 | $1\times10^5$ |
| | $3\times10^5$ | $61\pm2$ | $3\pm2$ | 0 | $2\times10^5$ |
| | $2\times10^5$ | $42\pm4$ | $1\pm1$ | 0 | $3\times10^5$ |
| | $1\times10^5$ | $19\pm1$ | $1\pm1$ | 0 | $4\times10^5$ |
| | 0 | 0 | 0 | 0 | $5\times10^5$ |
| Rib 3 | | | | | |
| | $2\cdot5\times10^5$ | $78\pm12$ | $7\pm4$ | 0 | $2\cdot5\times10^5$ |

Total cell concentration in mixing experiments $= 5\times10^5$ cells/culture.

TABLE V.—*Effect of Normal Marrow Colony Forming Cells of Cells from a Leukaemic Patient During Remission Induction and Subsequent Relapse*

| Normal marrow cells | | Mix | Patient's cells | | |
|---|---|---|---|---|---|
| Cell conc. | Colony No. | Colony No. | Colony No. | Cell conc. | Sample |
| $5\times10^5$ | $177\pm20$ | 0 | 0 | 0 | |
| $4\times10^5$ | $157\pm10$ | $192\pm12$ | 0 | $1\times10^5$ | Remission |
| $3\times10^5$ | $147\pm7$ | $166\pm14$ | 0 | $2\times10^5$ | induction: |
| $2\times10^5$ | $113\pm16$ | $111\pm3$ | 0 | $3\times10^5$ | |
| $1\times10^5$ | $53\pm9$ | $53\pm5$ | 0 | $4\times10^5$ | Peripheral blood* |
| 0 | 0 | 0 | 0 | $5\times10^5$ | |
| $2\cdot5\times10^5$ | $162\pm4$ | $59\pm5$ | $3\pm1$ | $2\cdot5\times10^5$ | Relapse: bone marrow† |

* Differential leucocyte count showed 92% lymphocytes, 8% neutrophils.
† Bone marrow sample approximately 80% replaced with blast cells.

with 3 different rib marrows, and Table IV gives these results in full. Clearly, the inhibitory effect of the leukaemic cells was similar on all 3 rib specimens. During the achievement of haematological remission this patient's (W.McM) peripheral blood leucocytes were again tested against normal marrow. At this time his peripheral blood differential consisted largely of lymphocytes; no blast cells or abnormal monocytes were present and the cells that were present had no inhibitory effect, although when his condition subsequently relapsed his cells inhibited colony growth (Table V).

### DISCUSSION

Peripheral blood leucocytes from normal individuals (which did not themselves give rise to granulocyte macrophage colonies in agar culture) caused only a slight and variable inhibition of colony formation by marrow cells cultured with them (Table I). Whenever bone marrow cells from normal individuals were cultured together the number of colonies obtained was that expected from counts made when the marrows were cultured separately (Table II). In contrast, peripheral blood or bone marrow cells from 7 of 9 patients with acute myelomonocytic leukaemia whose cells did not themselves give rise to colonies, caused a marked inhibition of colony growth by normal bone marrow cells (Table III). While one of the patients (B.S), whose sample did not cause this inhibition, had only 13% abnormal cells in his peripheral blood, his marrow (the sample tested) was almost completely replaced with blast cells. The other patient (R.G.) had a clearly abnormal differential and neither could be distinguished from the other on the grounds of clinical course or the morphological characteristics of their cells.

With those cases in which sufficient material was available to study various proportions of normal to leukaemic cells, inhibition could be shown when the ratio of leukaemic to normal was as low as 1 : 4. This is in contrast to the inhibitory effect of most normal peripheral blood specimens, which caused inhibitions only when the ratio of peripheral blood to marrow cells was 4 : 1 (Table I). Further evidence to support an association between this phenomenon and the leukaemic state was provided by studies using cells from a patient at different stages in his illness (Table IV and V). In this case cells were inhibitory at presentation, became non-inhibitory during the achievement of remission and became inhibitory again with relapse. Inhibitory effects were coincident with the presence of abnormal cells in the test samples.

While several authors have reported negative results in similar types of study (Greenberg, Nichols and Schrier, 1971; Robinson, Kurnick and Pike, 1971), Bull *et al.* (1973) found that in methylcellulose tissue cultures of marrow from patients with acute myeloid leukaemia receiving treatment, the presence of more that 20% of blast cells was frequently associated with colony counts below the normal range. They also found colony counts above normal when the proportion of blast cells was below 20% and further work is required to establish if these findings represent a direct effect of leukaemic cells on normal colony forming cells or are due to changes in colony forming cell kinetics resulting from a more generalized disturbance of marrow function. The variation of results may be due to the precise cultural technique used. Our culture system used conditioned medium rather than feeder layers (as by Greenberg *et al.* 1971 and by Robinson *et al*, 1971) as the source of colony stimulating factor which may have provided less suitable conditions for human colony growth. This possibility is suggested by two observations: (i) We seldom find any colonies growing in cultures of normal human peripheral blood, whereas others using feeder layer plates have reported the growth of small numbers of colonies (0–10 per $10^6$ cells) from this source (Kurnick and Robinson, 1971; Moore *et al.*, 1974); and (ii) out of marrow from 36 subjects with acute myelomonocytic leukaemia taken before the start of therapy, we were unable to grow colonies in 23 cases (64%). Moore *et al.* (1974), in a study of 108 cases of acute myeloid leukaemia, reported complete failure of growth in only 12% using cultures stimulated by feeder layers. However, these authors found that marrow from another 47% of patients gave rise to the growth of small clusters of 3–20 cells. It is possible that our 64% of non-growers are equivalent to the non-growers plus small cluster growers (59% of the total) described by the Melbourne group.

A suboptimal system for colony growth may be one in which colony growth is less sensitive to stimulating factor, or, alternatively, one in which colony growth is more sensitive to colony inhibiting factors. Colony formation in agar is dependent upon a complex medium and an unknown number of products from the heterogeneous cell populations included in the culture. With the exception of the colony stimulating factor (Metcalf, 1973), no clear distinction between basic nutritional factors and specific cell regulating factors has yet been achieved. It is known that normal blood and bone marrow cells can produce both colony stimulating and colony inhibiting factors and that stimulating factors originate mainly from low density glass-adherent cells (Haskill, McKnight and Galbraith, 1972; Moore, Williams and Metcalf, 1973; Messner, Till and McCulloch, 1973) of the monocytic type (Golde, Finley and Cline, 1972; Chervenick and Lo Buglio, 1972), whereas inhibiting factors mainly originate from high density cells (Haskill *et al.*, 1972) mainly of polymorphonuclear type (Paran, Ichikawa and Sachs, 1968 and Shadduck, 1971).

In the present work these complexities of interacting cell populations have been multiplied by the mixing together of cells from different individuals. In the experimental animal, genetically determined colony forming unit (CFU) repression has been demonstrated by McCulloch and Till (1973) and non-syngeneic stem cell inactivation seen in the mouse spleen colony technique (Petrov, Seslavina and Pantelejev, 1968). However, no loss of colony forming potential was noted in our hands from 6 co-cultures of marrows from different donors and we see little point in speculating about the mechanism of leukaemic inhibition of normal colony growth. Nevertheless, we feel that the phenomenon is more than a laboratory artefact and we present the data in the hope that other groups may attempt or re-attempt similar studies. The identification of cell inhibitory factors and their source could be a step in understanding the pathogenesis of marrow suppression in leukaemia.

We are indebted to Professor M. G. Nelson, Dr J. H. Robertson and Dr W. G. Wade for allowing us to study material from patients under their care, to Mr H. M. Stevenson for providing rib samples and to Mrs H. Jess and Mr J. G. Muldrew for technical assistance.

This work was carried out while T.C.M.M. was in receipt of a Royal Victoria Hospital, Belfast, research fellowship, with support from the Northern Ireland Leukaemia Research Fund.

### REFERENCES

Bradley, T. R. & Metcalf, D. (1966) The Growth of Mouse Bone Marrow Cells *in vitro*. *Aust. J. exp. Biol. Med. Sci.*, **44**, 287.

Bradley, T. R. & Sumner, M. A. (1968) Stimulation of Mouse Bone Marrow Colony Growth *in vitro* by Conditioned Medium. *Aust. J. exp. Biol. Med. Sci.*, **46**, 607.

Bull, J. M., Duttera, M. J., Stashick, E. D., Northup, J. Henderson, E. & Carbone, P. P. (1973) Serial *in vitro* Marrow Culture in Acute Myelocytic Leukemia. *Blood*, **42**, 679.

Chervenick, P. A. & Lo Buglio, A. F. (1972) Human Blood Monocytes: Stimulators of Granulocyte and Mononuclear Colony Formation *in vitro*. *Science, N.Y.*, **178**, 164.

Greenberg, P. L., Nichols, W. C. & Schrier, S. L. (1971) Granulopoiesis in Acute Myeloid Leukemia and Preleukemia. *New Engl. J. Med.*, **284**, 1225.

Golde, D. W., Finley, T. N. & Cline, M. J. (1972) Production of Colony Stimulating Factor by Human Macrophages. *Lancet*, ii, 1937.

Haskill, J. S., McKnight, R. D. & Galbraith, P. R. (1972) Cell—Cell Interaction *in vitro*: Studied by Density Separation of Colony Forming, Stimulating and Inhibiting Cells from Human Bone Marrow. *Blood*, **40**, 394.

Hullinger, L. & Blaztiovec, A. A. (1967) A Simple and Efficient Method of Separating Peripheral Blood Leucocytes for *in vitro* Studies. *Lancet*, i, 1304.

Kurnick, J. E. & Robinson, W. A. (1971) Colony Growth of Human Peripheral White Blood Cells *in vitro*. *Blood*, **37**, 136.

McCulloch, E. A. & Till, J. E. (1963) Repression of Colony-forming Ability of C57BL Hematopoietic Cells Transplanted into Non-isologous Hosts. *J. Cell comp. Physiol.*, **61**, 301.

McNeill, T. A. (1971) The Effect of Synthetic Double-stranded Polyribonucleotides on Haemopoietic Colony-forming Cells *in vitro*. *Immunology*, **21**, 741.

Messner, H. R., Till, J. E. & McCulloch, E. A. (1973) Interacting Cell Populations Affecting Granulopoietic Colony Formation by Normal and Leukemic Human Marrow Cells. *Blood*, **42**, 701.

Metcalf, D. (1973) Regulation of Granulocyte and Macrocyte-Macrophage Proliferation by Colony Stimulating Factor (CSF): A Review. *Expl Hemat.*, **1**, 185.

Moore, M. A. S., Spitzer, G., Williams, N., Metcalf, D. & Buckley, J. (1974) Agar Culture Studies in 127 Cases of Untreated Acute Leukemia: The Prognostic Value of Reclassification of Leukemia According to *in vitro* Growth Characteristics. *Blood*, **44**, 1.

Moore, M. A. S., Williams, N. & Metcalf, D. (1973) *In vitro* Colony Formation by Normal and Leukemic Human Hemopoietic Cells: Interaction Between Colony-forming and Colony-stimulating Cells. *J. natn. Cancer Inst.*, **50**, 591.

Morris, T. C. M., McNeill, T. A. & Bridges, J. M. (1974) Characteristics of Colony Growth from

Normal Human Bone Marrow. *J. clin. Path.*, **27**, 776.

PARAN, M., ICHIKAWA, Y. & SACHS, L. (1968) Feed Back Inhibition of the Development of Macrophage and Granulocyte Colonies II. Inhibition by Granulocytes *Proc. natn. Acad. Sci. U.S.A.*, **62**, 81.

PETROV, R. V., SESLAVINA, L. S. & PANTELEJEV, E. I. (1968) Stem-cell Inactivation in Mixed Spleen Cell Cultures. *Nature, Lond.*, **217**, 558.

ROBINSON, W. A., KURNICK, J. E. & PIKE, B. L. (1971) Colony Growth of Human Leukemic Peripheral Blood Cells *in vitro*. *Blood*, **38**, 500.

SHADDUCK, R. K. (1971) Granulocyte Stimulating and Inhibiting Activity from Neutrophils (PMN's): Possible Duel Feed Back Control of Granulopoiesis. *Blood*, **38**, 820.